# Discovering, Defining, and Summarizing Persistent Hotspots in SCORE Studies

**DOI:** 10.4269/ajtmh.19-0815

**Published:** 2020-05-12

**Authors:** Nupur Kittur, Carl H. Campbell, Sue Binder, Ye Shen, Ryan E. Wiegand, Joseph R. Mwanga, Safari M. Kinung’hi, Rosemary M. Musuva, Maurice R. Odiere, Sultani H. Matendechero, Stefanie Knopp, Daniel G. Colley

**Affiliations:** 1 Schistosomiasis Consortium for Operational Research and Evaluation, Center for Tropical and Emerging Global Diseases, University of Georgia, Athens, Georgia;; 2 Department of Epidemiology & Biostatistics, University of Georgia, Athens, Georgia;; 3 Division of Parasitic Diseases and Malaria, Centers for Disease Control and Prevention, Atlanta, Georgia;; 4 Swiss Tropical and Public Health Institute, Basel, Switzerland;; 5 University of Basel, Basel, Switzerland;; 6 Department of Epidemiology, Biostatistics and Behavioral Sciences, School of Public Health, Catholic University of Health and Allied Sciences, Mwanza, Tanzania;; 7 Mwanza Research Centre, National Institute of Medical Research, Mwanza, Tanzania;; 8 Neglected Tropical Diseases Unit, Center for Global Health Research, Kenya Medical Research Institute, Kisumu, Kenya;; 9 Division of Vector Borne and Neglected Tropical Diseases, Ministry of Health, Nairobi, Kenya;; 10 Department of Microbiology, University of Georgia, Athens, Georgia

## Abstract

The Schistosomiasis Consortium for Operational Research and Evaluation (SCORE) conducted large field studies on schistosomiasis control and elimination in Africa. All of these studies, carried out in low-, moderate-, and high-prevalence areas, resulted in a reduction in prevalence and intensity of *Schistosoma* infection after repeated mass drug administration (MDA). However, in all studies, there were locations that experienced minimal or no decline or even increased in prevalence and/or intensity. These areas are termed persistent hotspots (PHS). In SCORE studies in medium- to high-prevalence areas, at least 30% of study villages were PHS. There was no consistent relationship between PHS and the type or frequency of intervention, adequacy of reported MDA coverage, and prevalence or intensity of infection at baseline. In a series of small studies, factors that differed between PHS and villages that responded to repeated MDA as expected included sources of water for personal use, sanitation, and hygiene. SCORE studies comparing PHS with villages that responded to MDA suggest the potential for PHS to be identified after a few years of MDA. However, additional studies in different social-ecological settings are needed to develop generalizable approaches that program managers can use to identify and address PHS. This is essential if goals for schistosomiasis control and elimination are to be achieved.

## INTRODUCTION

The Schistosomiasis Consortium for Operational Research and Evaluation (SCORE; https://score.uga.edu) portfolio included several studies with multiyear mass drug administration (MDA) interventions for control and elimination of schistosomiasis.^1^ During the course of these multiple studies in different settings, it was observed that even with well-implemented MDA programs, there often remained locations in which infection prevalence and/or intensity did not decline to anticipated levels or bounced back to high levels.^2^

In October 2015, a number of researchers involved in SCORE met to discuss potential next steps in defining and understanding these unexpected recalcitrant hotspots. The discussion addressed what to call these areas that persist in the face of continued interventions, how they should be defined, what was causing them, and what research might be conducted to better understand them. SCORE colleagues from the Schistosomiasis Control Initiative and other participants noted that they were also finding similar hotspots in their non-SCORE research and program monitoring.^3^ There was agreement that these areas represented critical failures to achieve programmatic goals and would require new approaches. Subsequently, SCORE adopted the term persistent hotspots (PHS) to refer to villages or communities that fail to respond adequately to multiyear interventions.

This article describes investigations of PHS that SCORE has conducted since that meeting. These have used data from SCORE’s large, multiyear field studies of gaining and sustaining control^4–6^ to describe the occurrence of PHS and develop spatial analysis and predictive models. In addition, SCORE has layered studies of factors that might be associated with PHS onto the gaining control studies and onto a study on approaches to the elimination of schistosomiasis on Zanzibar^7^ to attempt to explain why some villages respond to multiyear interventions and others do not. Finally, we discuss the implications of PHS for neglected tropical disease programmatic decision-making.

## PERSISTENT HOTSPOT EVALUATIONS USING DATA FROM THE GAINING AND SUSTAINING CONTROL STUDIES

SCORE’s largest field studies involved evaluating various regimens for gaining or sustaining control of schistosomiasis.^4,5^ The gaining control studies were conducted in Kenya, Mozambique, and Tanzania in areas starting with a prevalence ≥ 25% during eligibility testing, and included six study arms, each with 25 villages, that received different community-wide (targeting children and adults) or school-based (only including children) MDA regimens.^5^ The most intensive regimen was annual community-wide MDA. The least intensive regimen involved 2 years of school-based MDA and 2 years that were praziquantel holiday years–years when praziquantel MDA was not conducted. The sustaining control studies were conducted in areas with a 10–24% prevalence during eligibility testing and included three study arms, each with 25 villages.^5^ The most intensive treatment arm in the sustaining control studies received annual school-based MDA. The other arms received 2 years of school-based MDA, either alternating with praziquantel holiday years (biennial MDA) or two consecutive years of MDA followed by two holiday years. The study diagram for gaining and sustaining studies is shown in Supplemental Figure 1. Niger originally had both gaining and sustaining control studies, but the Niger studies had to be redesigned because of a failure to randomize appropriately and are not included in this article.^4^

### Exploring alternative definitions of PHS.

In a follow-up to the 2015 meeting, SCORE used data from the first 3 years of the Tanzania gaining control study to assess four different suggested approaches by which to define PHS.^8^ The approaches were 1) absolute percent change in prevalence, 2) percent change in prevalence, 3) change in World Health Organization (WHO) risk categories, and 4) change (absolute or percent) in both prevalence and intensity. The same dataset yielded different numbers of PHS depending on the approach used to define them. The absolute change in prevalence (approach 1) may be useful for defining PHS if the starting prevalence is relatively high and similar among targeted locations. It does not work well if the starting prevalence is low because the change in prevalence must be set very small or villages will need to decrease to zero or even negative levels of prevalence to avoid being categorized as PHS. Defining PHS based on WHO risk categories (approach 3) is problematic because these WHO risk categories encompass wide prevalence ranges, with a ≥ 50% prevalence denoted as high risk, 10–49% as moderate risk, and < 10% as low risk.^9^ Thus, a village starting at a very high prevalence or at the high end of moderate risk could have a substantial decrease in prevalence, yet still be categorized as a PHS. In most places, we believe using the percent change in prevalence (approach 2), and incorporating intensity data when it is available (approach 4), might provide the best working definition of PHS until additional data and studies become available to help define PHS in ways that are most useful programmatically.

### Persistent hotspots in the final analysis of the gaining and sustaining control studies.

**Figure 1.**
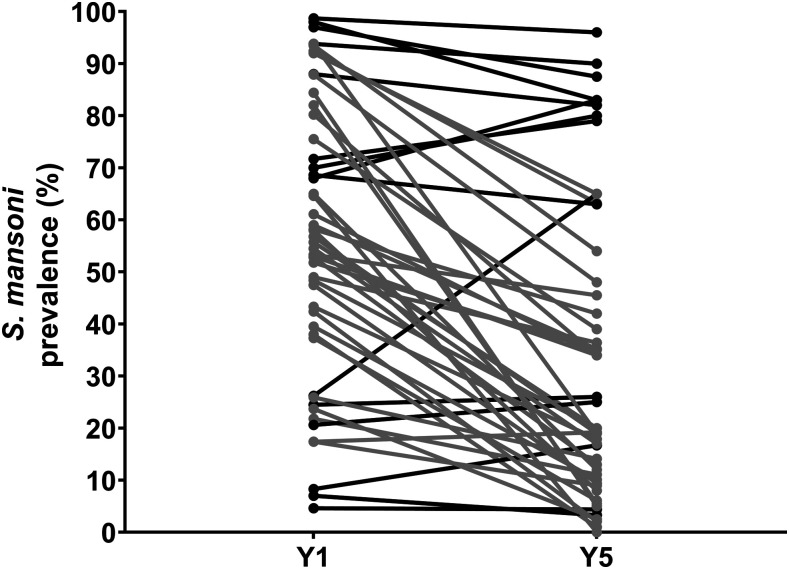
Line graphs showing prevalence at baseline (Y1) and final survey (Y5) in individual villages in study arms that received 4 years of annual school-based treatment in the SCORE Kenya and Tanzania gaining control studies. Gray lines depict villages that showed ≥ 35% reduction in prevalence, whereas black lines depict villages with < 35% reduction in prevalence from baseline to year 5. This figure is a composite of data presented in Ref. 2.

By the conclusion of the 5-year SCORE studies of gaining or sustaining control, the overall burden of schistosomiasis had declined in all studies and all study arms–whether MDA was provided as community-wide or school-based treatment, and whether it was provided every year for 4 years or twice in the 4 years (Supplemental Figure 2). However, there was a wide spectrum of village-level response to MDA within each of the study arms, and schistosomiasis prevalence and intensity failed to decrease or even increased in some villages.^2^ Figure 1 presents an example of this variability in response to MDA. Among villages in Kenya and Tanzania gaining control studies that received 4 years of annual school-based treatment, most villages, shown in gray, showed a substantial decrease in prevalence but some, shown in black, showed only slight decreases or even an increase in prevalence over the study period.

For exploratory analyses to better understand PHS, we defined PHS as villages that failed to achieve at least a 35% decrease in prevalence relative to baseline and/or a 50% decrease in infection intensity relative to baseline after 4 years of MDA, either annually or twice in 4 years,^2^ as an indication that schistosomiasis is not coming under control.

In the two sustaining control studies, between 30% and 60% of villages met our definition of PHS at Year 5. In the gaining control studies, PHS findings differed by country. In Kenya, 20–50% villages were PHS. In Mozambique, 65–80% of villages were PHS, but arms with annual treatment (i.e., four treatments during the study) had fewer PHS than arms that received treatment only twice over the 4 years. In Tanzania, in Year 5, all study arms had more than 60% PHS.

Our results indicate that PHS occur both in areas that start out with a relatively low prevalence among schoolchildren (10–24%) and those that start out higher (≥ 25%). Whether a village will become a PHS cannot consistently be predicted based on baseline prevalence or the starting prevalence of heavy infections.^2^

**Figure 2.**
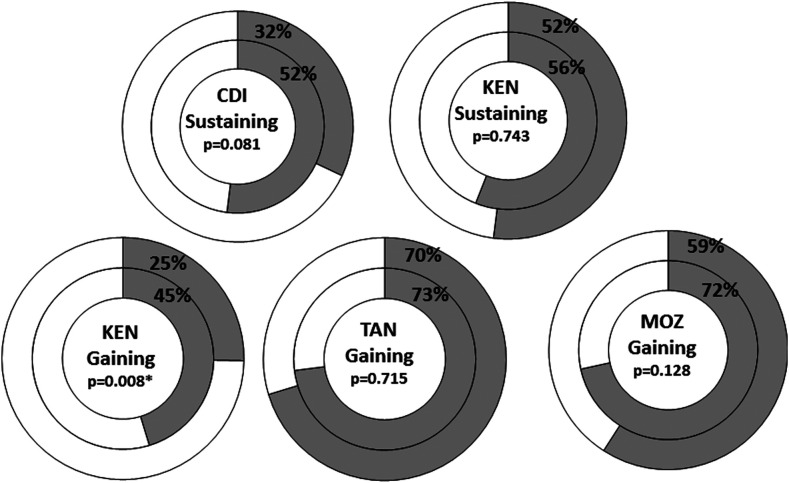
Donut graph depicting the proportion of persistent hotspots in SCORE gaining and sustaining control studies in Year 5 in study arms that had mass drug administration (MDA) twice in 4 years (inner ring) compared with study arms that had MDA every year (outer ring). χ^2^ *P*-value is indicated for each comparison. * Indicates statistical significance at *P* < 0.05. The data in this figure are re-graphed from data presented in Ref. 2.

Persistent hotspots occurred in all study arms, even in those with the most intensive treatment strategy—annual community-wide treatment. Study arms having four MDA treatments had fewer PHS than those that received only two MDAs over the study period (Figure 2). This result was statistically significant in the Kenya gaining control study (*P* = 0.008). Among study arms that received only two MDAs during the study, two MDAs followed by two drug holiday years resulted in more PHSs than MDAs without two consecutive drug holidays, which was also statistically significant in the Kenya gaining control study (*P* = 0.041).^2^

To examine the impact of coverage on a village becoming a PHS, for purposes of this analysis, we defined adequate coverage as ≥ 50% school-age children treated in Year 1 and ≥ 75% in subsequent years. Achieving these levels of MDA coverage did not seem to be associated with the likelihood of being a PHS. All villages in the Kenya sustaining control study met the definition for adequate coverage; nevertheless, almost 50% of the villages in each arm were categorized as PHS. In the other four studies, where this level of coverage was not reportedly uniformly achieved, the proportion of villages that were PHS was similar among villages that achieved adequate coverage and those that did not.^2^

### Predicting PHS with data from Kenya and Tanzania gaining control studies.

Early prediction of PHS within 1–3 years of starting MDA could potentially help programs use resources more efficiently and effectively. For example, efforts in PHS could be enhanced and less emphasis placed on villages that respond to MDA.

We used Year 1 (baseline) and Year 3 (after two MDAs) data from gaining control studies in Kenya and Tanzania to assess the performance of six modeling approaches to predicting PHS at Year 5 (final evaluation).^10^ Both of these studies took place in villages near Lake Victoria, where *Schistosoma mansoni* is endemic*.* Data from the Mozambique gaining control study were not included in this exploratory analysis because the study area was endemic for *Schistosoma haematobium*, a different species with a different biology.

The definition of PHS in this study was the same as in the aforementioned analyses. We applied six different predictive approaches, three of which were variants of conventional regression analysis and three of which were tree-based machine learning methods. A total of 10 predictors were considered in the full model, including prevalence, prevalence of heavy infections (≥ 400 eggs per gram [EPG]), infection intensity, and MDA coverage, each in Year 1 and Year 3.

Model performances were assessed using three scenarios. The first scenario used a subset of a country’s dataset for training and the remainder for validation, whereas the second scenario used one country’s dataset for training and the other country’s dataset for validation. In a third scenario, the data from Kenya and Tanzania were combined, and a portion of the combined dataset was used for training and the remainder for validation. Variables were then further evaluated for their importance in predicting PHS.

Overall, generalized linear models presented relatively stable performance compared with the tree-based machine learning methods. In the scenario using 70% of the Kenya dataset for training and 30% for validation, most models using both Year 1 and Year 3 data achieved more than 85% accuracy. Similar results were obtained using Year 3 data alone, suggesting that Year 3 data (after 2 years of MDA) may be helpful in identifying villages that are likely to be PHS in Year 5. Model performance was not as good with the Tanzania data.^10^

Despite both studies being conducted along Lake Victoria, models trained from one country’s dataset were not good at predicting the other country’s PHS, especially with the tree-based approaches. Using the combined Kenya–Tanzania dataset, performance of all models improved with the addition of a country factor, again indicating differences between countries.

The approaches leading to superior prediction performances were further investigated to identify important predictors for PHS. Mean infection intensity in the third year of MDA, the prevalence of heavy infections (≥ 400 EPG), and the prevalence of infection in the third year of MDA emerged as the most important variables in predicting PHS. The three combined contributed more than 60% of the overall prediction power across the tested models.^10^

Our investigation was limited to data from only two countries and the relatively few predictors that were available when the analyses were performed. Future efforts on data collection of village-level behavioral or environmental risk factors are likely to further improve the models. Similar studies in other areas might help in development of a series of models that could be applied across countries and contexts.

### Spatial analysis of hotspots using data from the Kenya gaining control study.

The Kenya gaining control study results were assessed for hotspots^11^ with spatial scan statistics,^12^ using SaTScan. For purposes of this analysis, a hotspot was identified by determining if schistosomiasis prevalence or intensity in SCORE study villages was randomly distributed over the study area. Note that this approach does not assume specific prevalence or intensity cutoff values in defining whether a village is a hotspot and does not require persistence of high prevalence or intensity over time.

The analyses found a spatial cluster of villages with a significantly higher schistosome prevalence or intensity than those outside the cluster (Supplemental Figure 3). The Kenya hotspot area had a consistent location and size across four disease measures (prevalence, mean intensity, median intensity, and high-intensity prevalence) and the three age-groups (5- to 8-year-old children, 9- to 12-year-old children, and adults aged 20–55 years) at baseline. In subsequent years, the location of the hotspot remained largely consistent, but the size varied by outcome and age-group. Further analyses found that baseline prevalence and high-intensity infections reliably predicted future prevalence.

Additional SCORE datasets, as well as those from other studies of schistosomiasis, could be used to explore the generalizability of our results to other endemic and non-endemic areas and spur discussions on how they might be used for programmatic decision-making. The use of spatial modeling to predict hotspots is also being studied for other diseases.^13^ A growing body of literature promotes an approach using model-based geostatistics and exceedance probabilities for a given threshold to define hotspots.^13,14^ This approach is not dependent on the sample size of the study and allows the program to set a threshold and determine hotspot boundaries rather than to have the statistical algorithm and hypothesis testing determine the appropriate bounds.

## FACTORS CONTRIBUTING TO PHS

The reasons that some villages or communities remain PHS are not well understood. Factors contributing to PHS were examined in the areas in Kenya and Tanzania, where the gaining control studies had been implemented, and in Zanzibar.

### The Kenya and Tanzania factors study.

Following completion of the gaining control studies in Kenya and Tanzania, SCORE evaluated factors that might differentiate villages that did not show substantial decrease in *S. mansoni* prevalence despite repeated, high treatment coverage (i.e., PHS) from villages that responded to MDA by showing a substantial decrease in prevalence (responders). For this study, PHS were defined as villages with a starting prevalence of ≥ 50% that remained at a ≥ 25% prevalence at the end of the study (while responders reduced to a < 25% prevalence), or those with a starting prevalence of 25–49% that remained at a ≥ 10% prevalence at the end of the study (while responders reduced to a < 10% prevalence). Factors investigated included behaviors, such as water usage patterns, water contact, and open defecation; the level of sanitation within schools and households; and proximity to known transmission sites. The geographic distribution of schools, water contact sites, and waterbodies within the villages were documented and mapped.

In Kenya and Tanzania, structured questionnaires were used to collect qualitative data from schoolchildren, household heads, and head teachers of the primary schools. In addition, urine and stool samples were collected from 50 children aged 9 to 12 years and 50 adults in each village. In Kenya, the study included six PHSs and six responding villages; in Tanzania, there were four villages in each group.

In Kenya, as expected, the prevalence of *S. mansoni* by Kato–Katz was significantly greater in the PHS villages (43%) relative to the responding villages (20%). Schools in responding villages were associated with higher odds of using protected water sources than those from PHS villages, and the proportion of children from PHS villages accessing surface water daily was significantly higher than that in responding villages. With respect to sanitation, schools in PHS villages had a lower ratio of latrines to pupils than schools in responding villages. Only 33% of PHS villages met the WHO-recommended latrine–pupil ratio for boys, and 0% met the WHO-recommended latrine–pupil ratio for girls. In comparison, 100% and 67% of the schools in responding villages met the WHO-recommended latrine–pupil ratio for boys (1:30) and girls (1:25), respectively. Similarly, households in responding villages had reduced odds of latrine sharing compared with households in PHS villages (R. M. Musuva et al., manuscript in preparation).

In Tanzania, as expected, the baseline prevalence of *S. mansoni* determined by the Kato–Katz assay was also higher in PHS villages (44%), compared with 22% in responding villages. The proportion of schoolchildren always using a toilet was higher in responding villages (90%) than in PHS villages (81%), whereas the proportion of people using surface water sources for bathing was 76% in PHS villages and 65% in responding villages. For both PHS and responding villages, the proportion of households owning a latrine/toilet was high (> 80%), whereas the proportion of households practicing open defecation was relatively low (< 20%), with no significant differences between PHS and responding villages.

In Tanzania, 39 key informants were interviewed, and 16 focus group discussion sessions were held, with a total of 123 participants. Based on these discussions, potential contributors to sustained high schistosomiasis prevalence among PHS villages included poor leadership style, lack of or insufficient social engagement, little or lack of genuine community participation, and little motivation and commitment to schistosomiasis control programs. In both sets of villages, discussants said that schistosomiasis was not given priority as an important health problem compared with acute illnesses such as malaria (J. R. Mwanga et al., manuscript in preparation).

### Persistent hotspots in the Zanzibar elimination study.

The Zanzibar elimination study involved 90 shehias (the lowest level administrative unit in the Federal Republic of Zanzibar) on Unguja and Pemba, the two main islands of Zanzibar. Shehias were randomized to three study arms: biannual MDA, biannual MDA + snail control, and biannual MDA + behavioral intervention. The study started in 2012 and included 5 years of intervention, followed by a final parasitological survey in 2017.

In a study conducted in 2014, a hotspot was defined as a shehia with a *S. haematobium* infection prevalence of ≥ 15% in schoolchildren aged 9–12 years in at least one of the three cross-sectional parasitologic surveys conducted at the time–at baseline (2012) or in 2013 or 2014 follow-up surveys. Shehias with a prevalence of ≤ 5% in schoolchildren in all three parasitologic surveys were considered as low-prevalence shehias. Investigators compared characteristics in five hotspot shehias in Unguja with those of two low-prevalence shehias. Hotspot shehias had a considerably larger number of human water contact sites containing the intermediate host snail *Bulinus* spp., a lower number of water taps with a constant water supply, and a shorter distance from the school to the nearest human water contact site than low-prevalence shehias.^15^

When the data from the entire 5 years of the Zanzibar elimination study were available, we conducted a further evaluation. Because the definition of hotspot by Pennance et al.^15^ did not include a feature reflecting “persistence” and because also the definitions provided by Kittur et al.^8^ do not apply well to elimination settings due to the mostly very low number of infected individuals, a different definition for PHS was needed for the situation in Zanzibar. For this analysis, PHS were defined as shehias with a prevalence ≥ 10% at baseline (2012) and ≥ 5% in 2017 in 9- to 12-year-old schoolchildren or adults aged 20–55 years.

Applying this definition to data from schoolchildren, there were six PHS shehias in Unguja and three in Pemba, with all arms on both islands having at least one PHS. Based on data from adults, there were three PHSs in Unguja and Pemba, respectively (one PHS shehia in each arm on each island). There was one overlapping PHS for schoolchildren and adults in Unguja and Pemba, respectively; the remaining PHS differed by age-group.

Besides the local characteristics for hotspots identified by Pennance et al.,^15^ we identified additional risk factors for *S. haematobium* infection in adults in Zanzibar, when adjusting logistic regression models for gender, age, island, and study year. Adults using river water for washing, bathing, washing clothes, or drinking had significantly higher odds of infection than adults not using river water. Also, adults complying with praziquantel treatment had significantly lower odds of infection than adults not taking treatment. In two of the three hotspot shehias in Pemba in 2011 and in one of three PHS shehias in Unguja in 2017, the mean proportion of adults using river water was significantly higher than that of adults using river water in the general study population of each island, which might at least partially explain why these shehias were PHS. None of the three PHS shehias in Unguja and Pemba had significantly lower praziquantel compliance rates than the whole of the island in any year, with exception of one PHS shehia on Pemba in 2014.

The reasons for the existence of PHS are diverse and may vary from area to area. Clearly, programs aiming to achieve elimination as a public health problem and interruption of transmission need to pay high attention to these places.

## PERSISTENT HOTSPOTS: SUMMARY AND IMPLICATIONS

The finding that PHS are ubiquitous in the SCORE studies was unwelcome for two reasons. First, and most importantly, the presence of PHS in several countries suggests schistosomiasis control programs are not having desired impacts in many locations, thus leaving people in PHS subject to reinfection and continued morbidity. Second, the level of variability within each study arm of this near-scale operational research program greatly increased the design effect, reducing the ability to achieve statistical differences between arms. Nevertheless, the SCORE studies have provided an important contribution by capturing how common PHS are in schistosomiasis-endemic areas and pointing out that the average decline in prevalence and intensity—the typical measure of MDA success—often hides many villages with less than effective schistosomiasis control.

Based on our studies and analyses, we believe there are three issues that now need to be addressed more fully for schistosomiasis control and elimination programs to achieve the goals described by the WHO and in the 2020 roadmap. The first is to clarify how to define PHS for programmatic purposes. In our studies, we used several approaches (Table 1). It is likely that the optimal definition will be different for programs with different capacities and goals (control versus elimination) and in different areas such as rural, peri-urban, or urban settings. The reason for some flexibility in definition of a PHS was most obvious when comparing the SCORE gaining and sustaining studies with the SCORE elimination study on Zanzibar. As discussed,^8^ using a given percent change only was simply not workable when dealing in locations with a very low prevalence. In addition, the best working definition may be based on such factors as whether modifiable risk factors can be easily identified and the amount of resources available for more intensive interventions. The second is to determine how and when (e.g., after how many years of MDA) can PHS villages be identified by programs. The results of the modeling studies indicate that prediction of PHS before the third year of MDA is possible. However, the optimal model and best approach to data collection and analysis may vary by site. For example, models developed with Kenya data were not predictive for Tanzania and vice versa. Spatial analysis worked well for identifying clusters of high-prevalence and intensity villages in Kenya but worked less well in Tanzania. Thus, much additional work is needed to define cost-effective sampling and monitoring schemes. These will likely lead to what is now called “precision mapping.”^16^

**Table 1:** Definitions of PHS in SCORE field studies

SCORE study	Definition of PHS
Sustaining and gaining control studies	Villages that failed to achieve at least a 35% decrease in prevalence relative to baseline and/or a 50% decrease in infection intensity relative to baseline after 4 years of mass drug administration, either annually or twice in 4 years
Kenya and Tanzania factors study	Villages with a starting prevalence of ≥ 50% that remained at ≥ 25% prevalence at the end of the study while responders reduced to a < 25% prevalence, or those with a starting prevalence of 25–49% that remained at ≥ 10% prevalence at the end of the study while responders reduced to < 10% prevalence
Zanzibar elimination study	Shehias with a prevalence ≥ 10% at baseline and ≥ 5% at the end of the study

PHS = persistent hotspots; SCORE = Schistosomiasis Consortium for Operational Research and Evaluation.

Third, once a PHS is identified, the actions needed to convert it into a responding village need to be determined. It is practically impossible to implement a full set of interventions in all endemic areas, and the cost-effectiveness advantage of optimizing minimum-package interventions is lost in PHS foci. Better identification of PHS could lead to the ability to focus additional interventions within identified PHS areas. These include innovations to increase coverage and frequency of MDA, better deployment of water, sanitation and hygiene interventions, integrated vector management, and better mainstreaming of behavior change communication that targets policy-makers, leaders, implementers, and community members. Also, research should be conducted to determine if it is possible to dial back efforts in places that are responding to MDA without losing ground there, with resultant savings in personnel time and money. In this regard, the term “shrinking the map” has become popular and indicates how control programs might move forward in addressing PHS.

The finding of PHS in locations with a very low prevalence, where elimination of schistosomiasis is considered feasible, presents an additional challenge. Persistent hotspots could act as reservoirs for the reintroduction of transmission into locations that are either under reasonable control or have achieved elimination. If transmission continues in these areas, there is a risk that infected individuals from these areas could reintroduce transmission in areas already free of transmission, or individuals from schistosomiasis-free areas could acquire infections in these areas and inadvertently restart transmission. Hence, in places such as Zanzibar, where there were many shehias with very low or no *S. haematobium* infections in 2017 and few PHS shehias, interventions need to be adapted to the local micro-epidemiology if elimination is to be achieved.^6,17^

Through the work described in this article, the SCORE has increased recognition of the importance of PHS and the need to develop adaptive approaches to address them. The epidemiologic and modeling investigations implemented by SCORE provide a basis for future work to better understand and conquer the recalcitrant problem of persistent schistosomiasis transmission in the face of multiyear intervention.

## Supplemental figures

Supplemental materials
