# Supplementary material for: Discovering, Defining, and Summarizing Persistent Hotspots in SCORE Studies
Source: Am J Trop Med Hyg. 2020 May 12;103(1 Suppl):24–9. doi: 10.4269/ajtmh.19-0815 (PMC7351310; doi:10.4269/ajtmh.19-0815)

Supplementary Figure 1. Study arms and timeline for the SCORE studies of Gaining and Sustaining Control of schistosomiasis in sub-Saharan Africa. Abbreviations: CDI, Côte d'Ivoire; CWT or C, community-wide treatment; H, drug holiday – a year when no praziquantel MDA was provided; SBT or S, school-based treatment.

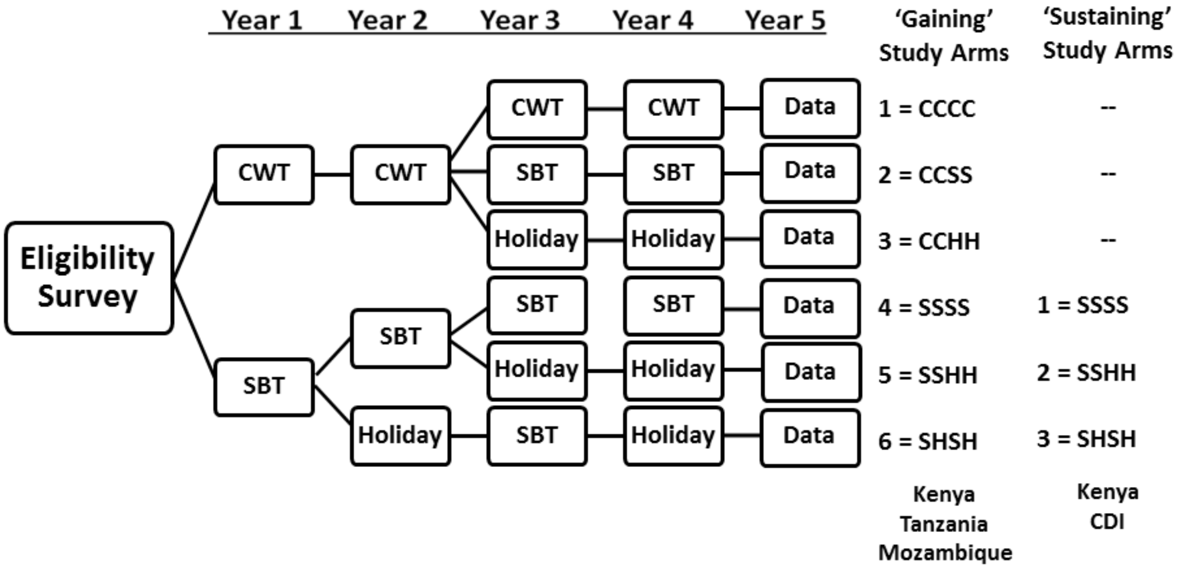

Supplemental Figure 2. Overall prevalence, by infection categories of light, moderate, and heavy intensity categories at Year 1 (baseline; Y1) and Year 5 (study endpoint; Y5) in the SCORE Gaining and Sustaining Control Studies. The study arms indicated are detailed in Supplementary Figure 1. Abbreviations: CDI, Côte d'Ivoire; C, communitywide treatment; H, drug holiday – a year when no praziquantel MDA was provided; S, school-based treatment; KEN, Kenya; MOZ, Mozambique; TAN, Tanzania.

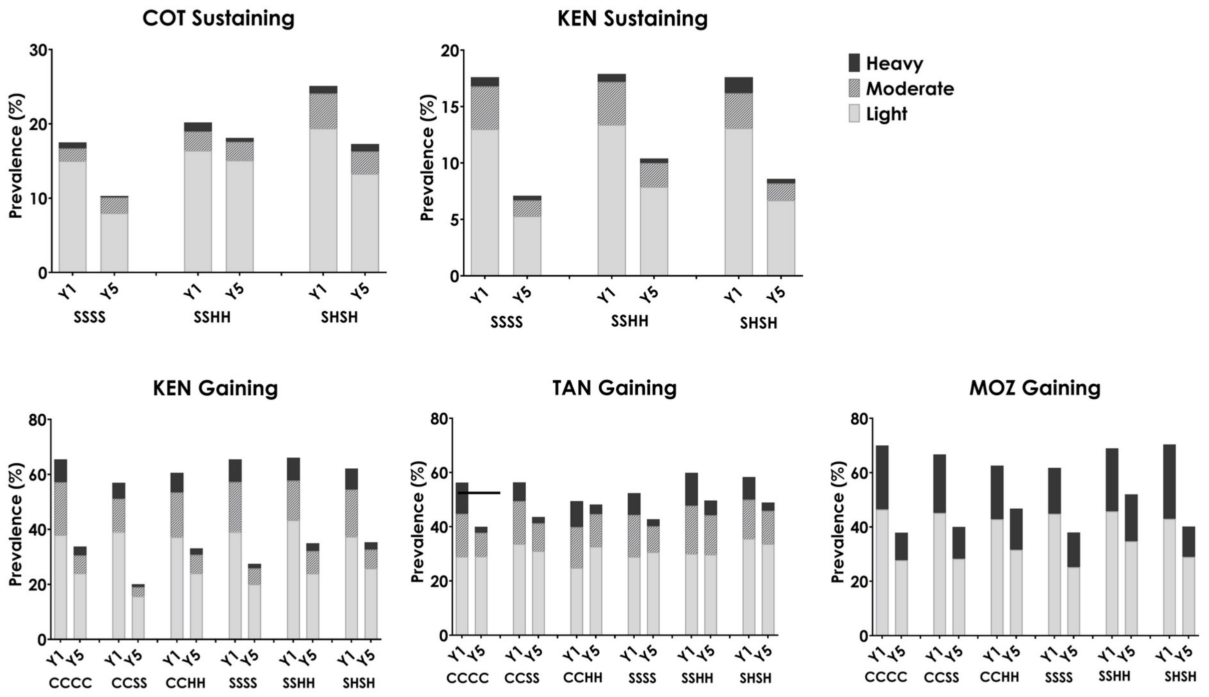

Supplemental Figure 3. Map of study villages in SCORE Kenya Gaining Study. Shapes denote the study arm and shading is according to village-level prevalence (percentage of participants with eggs in stool) for 9-12 year-old participants. The primary cluster from SaTScan analyses is outlined in blue. Notes in the lower left of the plot indicate the number of villages within the primary cluster, the relative risk (RR) of prevalence within the primary cluster compared to the prevalence outside the cluster, and the p-value (p) of the RR compared to a null value of one.

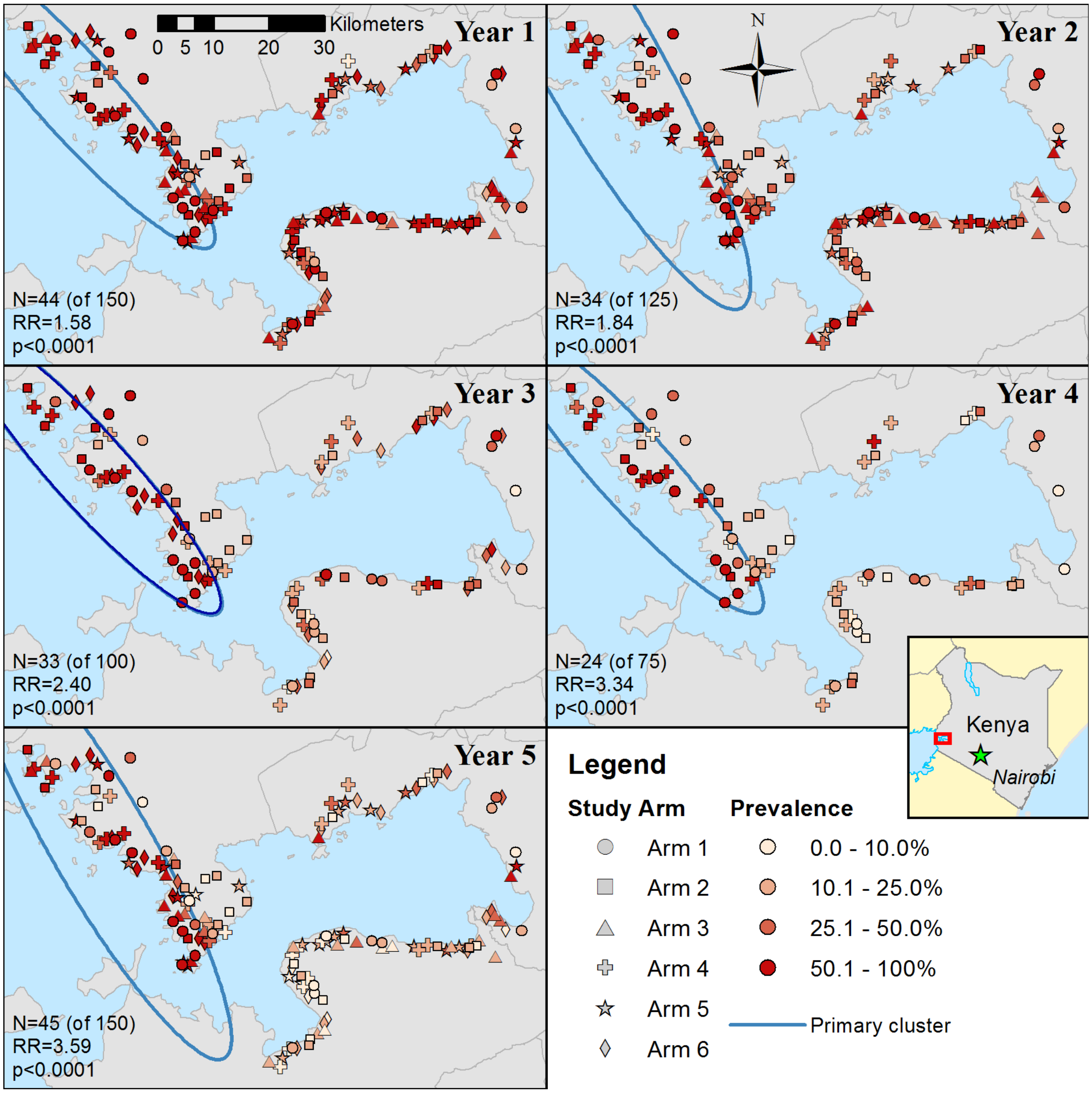

Supplement: Supplementary file 1 [file tpmd190815.SD1.pdf]
